# What stops us from eating: a qualitative investigation of dietary barriers during pregnancy in Punjab, Pakistan

**DOI:** 10.1017/S1368980021001737

**Published:** 2022-03

**Authors:** Muhammad Asim, Zarak H Ahmed, Amy R Nichols, Rachel Rickman, Elena Neiterman, Anita Mahmood, Elizabeth M Widen

**Affiliations:** 1 Department of Community Health Sciences, Aga Khan University, Karachi, Pakistan; 2 Department of Nutritional Sciences, College of Natural Sciences, University of Texas, Austin, USA; 3 School of Public Health and Health Systems, University of Waterloo, Canada; 4 Dell Pediatric Research Institute, University of Texas at Austin, 1400 Barbara Jordan Blvd., Austin, TX 78723, USA

**Keywords:** Nutrition during pregnancy, Food intake barriers, Morning sickness, Nutritional status, Qualitative research

## Abstract

**Objective:**

Adequate dietary intake during pregnancy is vital for the health and nutritional status of both mother and fetus. The nutritional status of reproductive age women in Pakistan is poor, with 14 % being underweight (BMI < 18·5) and 42 % experiencing Fe deficiency anaemia. This may stem from beliefs, practices and other barriers influencing dietary intake. This qualitative study seeks to determine which factors impact dietary intake during pregnancy in rural Punjab.

**Design:**

In-depth interviews and focus group discussions were conducted and then analysed using thematic analysis.

**Setting:**

Three purposively selected rural districts (Sahiwal, Okara and Pakpatan) with the highest prevalence of maternal and child malnutrition in the province of Punjab, Pakistan

**Participants:**

Mothers with children under age two (*n* 29) and healthcare providers with at least 5 years of experience working in the district (*n* 12).

**Results:**

We identified a combination of physiological, socio-cultural and structural barriers that inhibited healthful dietary intake during pregnancy. The primary physiological barriers to optimal dietary intake and dietary practices included food aversions and food cravings. Food classification, fear of a difficult childbirth, fear of high blood pressure and household food politics were the principal socio-cultural barriers. Additionally, two structural barriers, inadequate antenatal counseling and a lack of affordable food options, were identified.

**Conclusions:**

Our study demonstrates that complex barriers prevent pregnant women in the Punjab area from consuming adequate dietary intake and that antenatal health education programmes and structural interventions are needed to support healthful dietary practices during this critical period.

Pregnancy is one of the most nutritionally demanding phases in a woman’s life. Optimal dietary intake during pregnancy is critical to support the nutritional status of both mother and fetus^(1–4)^ and to reduce adverse pregnancy and birth outcomes, such as hypertensive disorders of pregnancy, preterm birth and low birth weight^(5–9)^. To support fetal growth, prevent the depletion of maternal nutrient stores and to ensure adequate nutritional status for breast-feeding, pregnant women require increased energetic intake in the second and third trimesters, as well as increased intake of specific nutrients, including Ca, Fe and folic acid^(10,11)^. Despite these recommendations, restricting dietary intake during pregnancy is common in Asian low- and middle-income countries (LMIC)^(10)^. Pregnant women in Asian LMIC consume less kilocalories, fats, proteins and carbohydrates than their counterparts in Caribbean and Central/South American LMIC^(11)^. To understand this phenomenon, various studies have highlighted socio-cultural barriers that hinder pregnant women’s food intake in Asian LMIC. These have included food aversions^(12,13)^, beliefs in associations between consumption of certain foods and delivery complications^(10)^ and cultural food classifications, such as those practiced in complementary and alternative medicine^(14–16)^.

This research has explored how socio-cultural norms affect food beliefs and perceptions about certain foods as beneficial or harmful. For example, stemming from Ayurvedic and Unani medical influences, a cultural construct of ‘hot’ and ‘cold’ foods is common in South Asian countries^(17,18)^ where every food item has innate properties to either heat or cool the body. Studies from Pakistan, Nepal and India have reported the belief that pregnancy is a state of excess heat in the body that can be offset by increasing consumption of ‘cold’ foods^(12,19,20)^. Violating these socio-cultural rules is believed to be associated with adverse pregnancy outcomes. For example in Pakistan, pregnant women classify fish, eggs, beef, eggplant, bitter gourd, greens, okra and dry fruits as ‘hot’ and associate their consumption with adverse pregnancy outcomes^(21–28)^. The effects of these foods are believed to be offset by eating ‘cold’ foods that include buttermilk, yogurt, oranges and cauliflower. Food classifications are not homogeneous across cultures and depict variations across regions.

These food-related beliefs may contribute to the suboptimal nutritional status of pregnant women and their infants observed in Pakistan. Recent estimates in Pakistan show a high proportion of reproductive age women with one or more nutrient deficiencies, including Fe deficiency anaemia (18 %) and vitamin A (27 %), Zn (22 %) and vitamin D (80 %) deficiencies^(32)^. Moreover, few women (29 %) reported taking Fe supplements for more than 3 months during pregnancy^(28)^. Suboptimal nutritional status among reproductive age women in Pakistan may have lasting effects on child health. Indeed, recent estimates in Pakistan show that over one in five children is low birth weight (22 %), and prevalence of stunting and wasting in children under age of five was 38 % and 7 %, respectively^(28)^.

Despite research from other LMIC reporting associations between socio-cultural influences and dietary intake in pregnant women, the nuances of these relationships have not been fully explored in Pakistan. Studies conducted in Pakistan have primarily adopted a quantitative methodology to evaluate pregnant women’s knowledge, attitudes and practices regarding dietary habits and micronutrient intake^(20,29)^. A qualitative approach can build on these insights to provide in-depth contextualised information on factors that impact human behaviour and dietary practices. This information is needed to inform and design health education programmes and structural interventions that facilitate nutritious eating habits. To address this gap, the current study was conducted to explore the factors that shape women’s dietary intake during pregnancy in Punjab, Pakistan.

## Methods

Considering the dearth of evidence related to dietary intake barriers in Pakistan, the current study was based on a qualitative descriptive research design that utilised in-depth interviews and focus-group discussions^(30)^. This design allowed us to interview those with differing experiences, including health care providers, which allowed flexibility and to generate a broader view of the complexities that mediate the dietary behaviour of women during pregnancy. Three purposively selected rural districts (Sahiwal, Okara and Pakpatan) in the province of Punjab, Pakistan, were chosen for the current study because they have the highest prevalence of maternal and child malnutrition in Punjab, Pakistan’s most populated province^(31)^.

### Study participants

The current study included two categories of participants: mothers and healthcare providers. In total, we conducted three focus group sessions with twenty mothers and a total of twenty-one individual interviews, nine with mothers and twelve with local healthcare providers (Table 1). For a better recall of the pregnancy experience, the eligibility criteria for selecting mothers required them to have been pregnant within the last 2 years. For a broad perspective, we selected three types of healthcare providers: community health workers from Pakistan’s Lady Health Worker (LHW) Programme^(32)^, midwives and general practitioners. The eligibility criteria for selecting healthcare providers required that they have at least 5 years of experience working within the district.

**Table 1:** Study participants (*n* 41)

Stakeholder	In-depth interview	Focus-group discussion	Total
Mothers	9	20 (3 sessions)‡	29
Lady health workers*	6	–	6
Midwives†	3	–	3
Female general practitioners†	3	–	3
Total	21	20	41

* Two per district.

† One per district.

‡ 6–7 Participants in each focus group, one focus group per district.

Since the research topic required participants to talk about intimate aspects of their lives, all participants were recruited through purposive sampling so that we could target information-rich cases. Initial contact was made with the local health counselor working within each district. These individuals served as entry points to the community and identified healthcare providers for the study. In turn, the healthcare providers helped recruit mothers to participate in the study. An equal number of women and healthcare providers were selected from each district (Table 1).

### Interview guide development

Semi-structured interview guides were developed for interviews with mothers and healthcare providers (see online supplemental materials). These guides were formulated after an extensive literature review using different keywords related to the socio-cultural determinants of maternal diets in LMIC, i.e. food, dietary or nutritional barriers during pregnancy. The guides were pilot-tested with mothers and healthcare providers before data collection. Moreover, the interview guides were also periodically updated over the course of the study as more details about the community were discovered.

### Data collection

Data were collected from January to March 2018. We first conducted twenty-one semi-structured in-depth interviews with nine mothers and twelve healthcare providers followed by three focus-group discussions with twenty mothers (Table 1). Mothers were interviewed at the local ‘health house,’ a designated space within the community where they routinely visit to obtain basic healthcare services, contraceptives and child vaccinations. This neutral location was selected to provide a safe environment for mothers to share their personal experiences. All healthcare providers were interviewed at their workplaces. The interviews were conducted face-to-face in Urdu by the first author (MA, PhD candidate), who is most familiar with the local culture, alongside two female social science research assistants trained on the study protocol and research methodology. The research assistants were recruited due to cultural norms in rural Punjab where unaccompanied interactions between males and females are restricted.

After completing the in-depth interviews^(21)^, we used the results to further refine our semi-structured interview guide for the focus group discussions. A total of three focus group discussions were held (one in each district) with 20 mothers who were not part of the in-depth interviews. Each session hosted 6–7 participants, and the discussions were moderated by the first author with the help of two research assistants. The sessions focused on discussing common themes that arose during the in-depth interviews, including food meanings and classifications, household politics and behaviour related to seeking healthcare. The sessions enabled exploration of the range of opinions within the group, generated a holistic view and verified any contradictory findings from the interviews.

The in-depth interviews lasted 25–30 min and focus group discussions were 40–50 min. All individual interviews and focus group discussions were audio-recorded, and written field notes were taken by the research assistants to track participants’ nonverbal and verbal communication, including body language and moments of hesitation. Recordings were immediately transcribed verbatim into the English language by the MA and were counter-checked by ZHA to ensure data authenticity. A debriefing session followed each interview and focus group discussion to allow the research team to reflect upon the interview process, examine findings and resolve discrepancies. Data collection was concluded after all authors agreed that the interview discussions and data collected had reached a point of information saturation.

### Data analysis

The data analysis process included an intensive review of all transcripts and field notes by two co-authors (MA and ZHA) through manual thematic analysis. To ensure data quality, all transcripts were verified a second time by the research assistants using both written notes and audio recordings. The inductive method was used to formulate study themes; this approach employs a detailed reading of raw data to assemble concepts, themes and interpretations from participants’ responses^(33)^. Two co-authors generated an exhaustive list of themes through a detailed review of the transcripts. These themes were used to develop a theoretical matrix reviewed by all authors and from which several sub-themes emerged. Then, transcripts were reviewed to extract quotes corresponding to each sub-theme. Discrepancies during this process were resolved with the mutual consent of co-authors through triangulating and validating the narratives of different participants (mothers, healthcare providers and field observations). Similar codes or recurring participant statements were omitted during manuscript preparation.

## Results

**Fig. 1:**
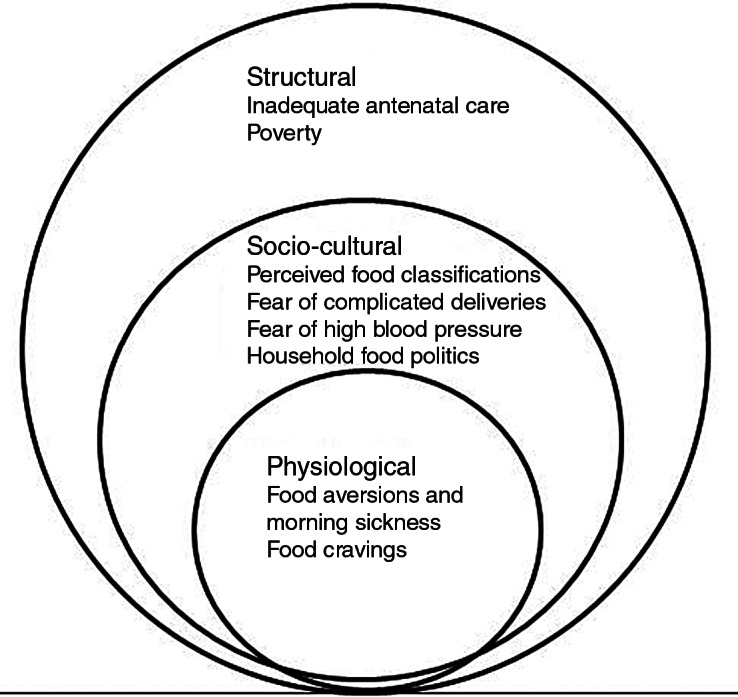
Socio-ecological model highlighting major food intake barriers identified in the current study

After analysing the results of in-depth interviews and focus group discussions, three broad categories of food intake barriers emerged: physiological, socio-cultural and structural barriers (Fig. 1). The descriptive characteristics of mothers included in study are presented in Table 2.

**Table 2:** Descriptive characteristics of mothers included in study (*n* 29)

	Frequency	Percentage
*Education		
Less than primary education	13	44·8
†Primary and above	16	55·2
Age		
Up to 25 years	12	41·4
26–35 years	10	34·5
36 years and above	7	24·1
Number of children		
2–3	17	58·6
4 and above	12	41·4

* Taken from mother’s recall.

† Five grade and above.

### Physiological barriers

Physiological barriers to food intake refer to an individuals’ bodily reactions to certain food items. These consisted of food aversions, including those associated with morning sickness, and food cravings.

### Food aversions and morning sickness

During our interviews and focus group discussions, several women reported changes in dietary intake due to food aversions caused by morning sickness. A mother of three stated:

‘During pregnancy, I suffered from morning sickness and had to completely change my eating habits. I found it difficult to consume my routine diet, such as milk, bread, and cooked foods.’ (Mother, FGD)

This response was reinforced by another participant who reported antenatal food avoidance due to a fear of vomiting:

‘I did not eat properly for several weeks because I was afraid I would vomit. I only ate light foods, such as biscuits [i.e. plain cookies] and crackers. I lost a lot of weight because of this.’ (Mother, In-depth Interview)

In rural Punjab, the customary diet includes roti (flatbread) accompanied by a curry of either vegetables or lentils. Interview responses highlighted that pregnant women were often averse to this staple meal due to the effects of nausea or morning sickness:

‘I cannot tolerate the smell of salan [curry] or roti [bread]…When I was pregnant, I would not even go near the kitchen because I felt the smell would make me vomit.’ (Mother, FGD)

These responses indicate that commonly consumed foods induced food aversions or nausea for many participants. As a result, traditional meals were avoided in favour of less nutrient-dense foods that may curb nausea, such as biscuits and crackers, and limiting overall dietary diversity and energetic intake.

### Food cravings

Interviews revealed that women craved a variety of sour, sweet and salty foods with preferences varying across pregnancy. Other cravings included non-edible or non-nutritive items, such as ice, chalk or dirt, a condition otherwise known as pica. For example, a rural LHW stated from her field experience:

‘Some pregnant women develop a craving to start eating clay and coal.’ (LHW, In-depth Interview)

A mother corroborated this observation:

‘When I was pregnant, I craved eating baked clay, particularly in the last trimester of the pregnancy. My mother-in-law had forbidden me to do so, but I was eating it secretly.’ (Mother, In-depth Interview)

These cravings for non-food items or foods with low nutrient density may displace intake of more nutrient dense foods and also impact overall dietary diversity.

### Socio-cultural barriers

The socio-cultural barriers presented here refer to beliefs and values related to food consumption that may limit pregnant mothers from consuming a well-balanced diet that meets macronutrient and micronutrient needs during pregnancy. The current study identified four such barriers: cultural food classifications, fear of a difficult delivery, fear of high blood pressure and household food politics.

### Food classifications

Our study revealed that women in rural Punjab were highly concerned about consuming certain foods in these categories, which guided food choices during pregnancy. For example, during the first two trimesters women consumed more items classified as ‘cold’ foods, such as lassi (a drink made from yogurt), raw butter, oranges, cucumber and cauliflower, whereas ‘hot’ food items, such as clarified butter, and dry fruit (dry dates, raisin, and coconut), were more often chosen during the third trimester:

‘I tried to consume Thanda [“cold”] items early in my pregnancy as it helps with morning sickness. The body is already producing heat during this time, so we need to cool it down by eating items like yogurt, lentils, and vegetables. Later on, when we are getting ready to give birth, the body changes. During this time, we can eat more hot items, such as animal proteins and fats. These help with the delivery of the baby.’ (Mother, FGD)

This dietary regulation is reinforced by close relatives and traditional birth attendants. In particular, the participants noted that the role of the mother-in-law can be pivotal in emphasising the importance of ‘hot’ or ‘cold’ foods:

‘I wished to eat more protein-rich foods, but my mother-in-law did not allow me to eat these items because they were “hot” in essence. She [mother-in-law] said that the Dai [traditional birth attendant] had told her not to let me eat any “hot” foods as they may cause a miscarriage.’ (Mother, FGD)

In addition to the concept of ‘hot’ and ‘cold’ foods, participants also discussed ‘hard’ foods that referred to items considered difficult to digest. According to a rural LHW:

‘Most of the mothers avoid consuming “hard” foods, such as chickpeas, red lentils, corn, and guava, during pregnancy because they believe these items may cause bloating, stomach pain, and negatively affect the fetus.’ (LHW, In-depth Interview)

The cultural pressure associated with these perceptions may prevent pregnant women from consuming a balanced diet that supports the nutritional requirements of pregnancy, particularly protein-rich foods.

### Fears of complicated deliveries

Our interviews indicated that some women associated the excessive consumption of food with a larger fetus and, consequently, a difficult delivery. Women who adhered to this belief consciously reduced the quantity of food they consumed in order to avoid complications that might occur from delivering a larger baby. This point was voiced by a mother of three:

‘I try and keep myself to having two small meals a day. If I eat more, my baby will become too big. I fear that if my baby is too large, I will not be able to deliver itx at home and will have to spend extra money visiting the hospital and getting a C-section.’ (Mother, In-depth Interview)

Another mother reported that she restrained herself from eating, despite feeling hungry:

‘In my third trimester, I felt hungry … I was afraid that by eating more I would increase the size of the fetus. I seldom ate three proper meals a day.’ (Mother, FGD)

Additionally, some women believed reducing food intake during pregnancy decreased the likelihood of Cesarean delivery. This surgical procedure must be carried out in a hospital and may be prohibitively costly, especially for rural, lower-income households. A rural midwife alluded to this point:

‘Women eat less food during pregnancy because they think that it will lead to normal delivery and they will not have to undergo C-section. Women here don’t have money to run their houses properly, how will they arrange the money for a C-section.’ (Midwife, In-depth Interview)

The belief that consuming an excessive amount of food during pregnancy leads to a larger fetus and difficult delivery may influence mothers to reduce their overall dietary intake, resulting in fewer nutrients available to support pregnancy and lactation.

### Fears of high blood pressure

Respondents held certain beliefs about the physiological consequences of consuming certain foods. During interviews, some women revealed perceptions that certain nutrient-dense foods, such as fats, meat, and eggs, may cause high blood pressure and actively restrained themselves from consuming these items. For example, a mother of four children explained:

‘When I was pregnant I only ate dal roti [bread with lentils] due to the fear of high blood pressure. Whenever I ate meat, fats, and fried food, I felt as if my blood pressure was rising.’ (Mother, FGD)

Such views may have originated from medical personnel, as indicated by a mother of two:

‘The doctor forbade me from eating food items that may cause high blood pressure…. that it would increase the risk of a bad pregnancy if I ate a lot of red meat or chicken.’ (Mother, In-depth Interview)

It is probable that this perceived fear, plus directions from care providers, family members, or friends, makes these women feel more vulnerable and presents a psychosocial barrier to eating more nutritionally varied foods, in this case animal proteins and accompanying micronutrients (e.g. Fe).

### Household food politics

The influence of household food politics on the diet of pregnant women was also discussed. Specifically, women seeking to supplement their diets with more nutritious foods were often prevented from doing so due to household food-sharing dynamics. Women living within such a system may be pushed toward eating a standardised diet, with little room for special requests:

‘When I was pregnant, I felt a lot of hunger and my mother-in-law used to say, ‘You are not special because you are pregnant. Be thankful to God, and eat whatever is available at home. I had six children and no one ever fed me anything special.’ (Mother, In-depth Interview)

This situation may be further exacerbated by household norms that often limit the social mobility of women, preventing them from making autonomous choices:

‘I was told by my mother-in-law to just eat dal [lentils] and roti [bread] and not to make any special requests. Sometimes I craved something sour or salty but I was not allowed to leave the house to go to the market alone. I was totally dependent on my mother-in-law for food…’ (Mother, In-depth Interview)

The role of the mother-in-law was described as mediating the dietary pattern of the pregnant mother. Aside from enforcing the household norms, mothers-in-law sought to maintain their hierarchical standing within the household by actively blocking preferential treatment for their pregnant daughters-in-law. This was described by a rural LHW:

‘When the new bride in my neighboring household became pregnant, her husband brought her jalebi [a sweet dish] because she had been craving it for days. This caused a fight to break out in the house…. I think she felt threatened because her son was bringing food for his wife and neglecting her [the mother].’ (LHW, In-depth Interview)

These responses indicate that food consumption decisions are not always in the hands of pregnant mothers. Despite an intention to eat more nutritionally dense foods, diverse diets, or to even respond to food cravings, the pressure of household food politics prevented some pregnant women from accessing the foods they wanted or needed.

### Structural barriers

Structural barriers to food intake refer to macro-level community factors, such as policies, practices, or procedures, that result in some people receiving unequal access to nutritious food. Two such barriers were identified in the current study: inadequate antenatal counseling and the unaffordability of nutritious food options.

### Inadequate antenatal counseling

Discussion with mothers in our study indicated that rural women have limited awareness, food literacy, and may lack motivation regarding the dietary or nutrient requirements for a healthful pregnancy. For instance, one mother reported:

‘I don’t know which foods are beneficial during pregnancy. I have four children and I don’t even care about what food I should consume during pregnancy. I eat whatever food is available at home. I do not take any supplements or tablets during pregnancy.’ (Mother, FGD)

This lack of nutritional awareness was compounded by women missing the opportunity to seek antenatal care because of poor experiences during prenatal care. A mother of two recalled:

‘During my first pregnancy, I was very keen to go to all my antenatal visits…. But when I reached the clinic … There were 50–60 women lined up outside waiting for the doctor. I waited 2 h for my turn. When I finally met the doctor, she spent a total of 2 min with me. She offered me no dietary advice and just gave me a prescription for a few multivitamins.’ (Mother, FGD)

It was not surprising to learn that, often, women in our sample only sought antenatal care when they experienced pregnancy complications. According to one rural GP, this visit was when many received their first antenatal nutritional guidance:

‘Some women come in the third trimester with complications, especially anemia and preeclampsia…. They don’t care about the diet and only demand multivitamin tablets and injections for a quick recovery from anemia. We give some multivitamins and folic acid tablets and instruct them to eat more fruits, vegetables, milk, and meat.’ (GP, In-depth Interview)

Antenatal care typically provides an opportunity to counsel women about the importance of consuming healthy dietary patterns and nutrient rich foods during pregnancy. However, counseling and dietary recommendations were overlooked in our study area. As such, most women we interviewed were unaware of the importance of a diverse diet and increased energy requirements during this time.

### Unaffordability of nutritious food options

Our participants reported that certain food items, such as meats, fruits and vegetables, are not affordable for low-income families. According to one mother of four:

‘My husband earns 600 rupees (4USD) per day and I cannot buy fruit, milk, and meat on this limited budget…. We could only afford to eat basic items, such as dal roti [bread with lentils], during pregnancy.’ (Mother, In-depth Interview)

In the absence of adequate nutrition, per one LHW, women may be encouraged to consume an extra meal plus multivitamins each day:

‘Most of the households, especially in rural areas, cannot afford a nutritious diet, i.e. milk, fruits, dry fruits, and meat. In this situation … I instruct them to take an extra meal a day and eat more curry [lentils and vegetables] with bread. I give some multivitamins and folic acid tablets to increase hemoglobin level.’ (LHW, In-depth Interview)

Depending on economic conditions, food availability and affordability and adequate nutritional guidance, adding extra dietary food staples and supplements may not be an attainable solution in rural Pakistan.

## Discussion

Adopting a broad-scoped qualitative methodology the current study identified the right barriers preventing women in rural Punjab from consuming nutritionally dense foods and achieving dietary diversity during pregnancy. These included two physiological barriers, food aversions and food cravings, plus four socio-cultural barriers, food classification, fear of difficult delivery, fear of high blood pressure and household food politics. Additionally, two structural barriers were identified, inadequate antenatal care and the lack of affordable nutritious food (Fig. 1).

### Physiological barriers

It is well established that nausea and/or vomiting in early pregnancy are common conditions affecting 35–91 % of pregnant women^(11,34–47,48,49)^. This may result in altered dietary intake and food preferences^(34)^. A study by Verberg *et al.* (2015) indicated that the effects of morning sickness may be more pronounced in Pakistan and India compared with Western countries. These symptoms have been attributed to causes ranging from Zn deficiency to psychosocial stress^(50)^. Women affected by morning sickness may find certain foods intolerable and can face unusual food cravings^(51)^. Our interviews indicated that some women found their routine diets of daal and roti intolerable and others craved non-food items, such as clay. This practice, called geophagy, is a form of pica^(52)^. The association between pica and nutrient deficiencies is ambiguous. There is evidence that nutrient deficiencies, such as Zn and Fe, may lead to pica, or cravings for nonnutritive items^(35,36)^. However, consumption of non-nutritive items like clay, a practice known as geophagy, can lead to binding of certain micronutrients, such as Fe or Zn, that may lead to deficiency. Several studies observed strong associations between geophagy and Fe deficiency anaemia^(53,54)^.

### Socio-cultural barriers

Interviews in the present study revealed that mothers classified food items into three categories, ‘hot,’ ‘cold’ and ‘hard’ foods, and regulated their dietary intake based on the rules of these classifications. This pattern is common in South Asia, where influences from Ayurvedic and Unani humoral medicine prevail, and a balance of ‘hot’ and ‘cold’ is believed to be necessary to maintain health^(55)^. Since the first trimester of pregnancy is believed to generate a state of ‘heat,’ it is important to balance the body by consuming ‘cold’ foods, such as yogurt, milk and buttermilk^(19,56,57)^. This pattern is reversed in the third trimester where the pregnant body is thought to be ‘cool.’

While the concepts of ‘hot’ and ‘cold’ foods are widespread throughout South Asia, the underlying criteria for classifying foods are often not clear. Studies indicate that considerable variation exists in this respect not only between different countries but also within regions. Responses from our study reveal that animal protein items, such as poultry, fish, meat and eggs, are considered ‘hot’ foods and are therefore avoided during the early stages of pregnancy. Lack of adequate protein foods, such as meats, may limit availability of essential amino acids, vitamins B_6_ and B_12_, Fe and Zn during this critical phase of fetal development^(53)^. Moreover, given the high prevalence of Fe deficiency anaemia among women of reproductive age in Pakistan (18·2 %), intake of protein and Fe-rich foods that are culturally appropriate for the stage of pregnancy (i.e. lentils and beans in early pregnancy) are important to address during nutritional counseling and interventions^(27)^. Foods identified as ‘hard’ during interviews were fibrous, including chickpeas, corn, guava, carrots and lentils. Our interviews indicated that mothers found these foods difficult to digest and felt that they caused stomach pain and bloating. This finding is supported by research from Ethiopia which showed that mothers perceived ‘hard’ foods as difficult to digest and associated with abdominal distension, bloating and difficult deliveries^(58)^.

Our interviews also revealed that mothers believed that consuming more food during pregnancy would increase the size of the fetus and subsequently lead to a complicated delivery. It is possible that this belief was fueled by a rise in cesarean deliveries in Pakistan from 2·7 % in 1991 to 22·3 % in 2017^(28)^. For instance, interviews also indicate that some women view hospital deliveries as a financial burden on their families and strive to eat less food to limit the size of the baby and allow for a home birth. This finding is supported by several studies from other LMIC^(59–61)^, including Bangladesh, Ethiopia and Zambia, which highlight the link between poverty and preference for home deliveries. The potentially detrimental consequences of such associations may be countered through the involvement of LHW who can incorporate nutrition counseling as part of their door-to-door visits.

Household food politics refers to the conflict or competition over the control of food and its sources at the household level^(62)^ and is another socio-cultural barrier to adequate maternal nutrition. More than half of the families in Pakistan live under an extended family, primarily with the husband’s parents and other siblings, in which members are expected to share food equally^(41)^. In rural joint families, tenets of food politics are usually enacted by the mother-in-law. As a newly inducted member in the household, the daughter-in-law is expected to slowly earn her place in the house by apprenticing under her mother-in-law^(63)^. As demonstrated in the current study, allowing the daughter-in-law to consume food items of her preference inverts this established power dynamic and may be met with restraint. Other studies from Africa, Asia and Latin America have also reported that mothers-in-law play an instrumental role in determining the diet of pregnant mothers, especially during pregnancy, childbirth and infant feeding^(64)^. Food politics may amplify effects of some of the other barriers identified in the current study. For example, the inability to access certain foods, such as citrus fruits, vegetables and breakfast cereals, may make women more susceptible to nausea and micronutrient deficiencies^(34)^. Greater awareness is needed regarding the implications of household food politics on the dietary practices of pregnant mothers. This can be addressed through awareness-raising campaigns that target mothers-in-law to encourage household decision-making that is sensitive to the dietary needs of pregnant mothers.

### Structural barriers

Studies have reported that the quality of antenatal care is poor in Pakistan^(65,66)^. Participants in our study reported that women in rural Punjab do not receive adequate maternity care, thus they rarely followed through with antenatal visits. Those who did attend their appointments received limited or no nutrition counseling. Evidence from Majrooh *et al.* (2014) confirmed this finding: only 56 % of pregnant women enrolled for antenatal care in Punjab and 33 % missed subsequent visits^(45)^. Moreover, Munawar emphasised that more than 52 % of participants reported feeling humiliated by health professionals during maternity care in Pakistan^(67)^.

These are notable findings, since a strong, supportive antenatal system could play a critical role in addressing many of the barriers identified in the current study. Antenatal counseling is one avenue for guidance of evidence-based nutrition information, emphasising the importance of consuming a diverse diet to support pregnancy. Counseling can provide mothers with the opportunity to assess the quality of their diets in ways other than culturally mediated food classifications^(68)^. This form of nutrition education addresses misconceptions about food and nutrition during pregnancy, such as the association between certain foods and heightened blood pressure or larger babies. Through nutrition consultations, modifications to everyday meals could help mothers avoid nausea-inducing foods while still meeting nutrient requirements. For instance, an emphasis can be placed on the consumption of smaller meals, more frequent meals, alternative sources of protein and hydration^(69)^.

Pakistan is an LMIC with almost one-fourth of the population living below the poverty threshold^(46)^. Some barriers to accessing nutritious foods are due to changes in Pakistan’s political and economic climate. Our study reported that families living in rural Punjab are unable to afford nutritious food in the form of fruits and vegetables. The inability to consume fresh produce restricted the consumption of vitamins A, C, folate and other B vitamins during critical phases of fetal development^(53)^. Addressing the barriers to fresh fruits and vegetables for marginalised segments of society requires large-scale structural changes, including greater income equality and land reforms. While a discussion of these is beyond the scope of this paper, short-term strategies can be devised to inform mothers on how to optimise their dietary intake amidst scarcity by creating recipes for affordable nutrient-dense foods.

### Strengths and limitations

This qualitative study uncovered multifaceted dietary barriers during pregnancy that have not been fully explored in Pakistan. A broad-scoped qualitative methodology enabled us to unearth eight unique physiological, socio-cultural and structural barriers. Since the current study was conducted in three purposefully selected districts of Punjab, our results may not reflect the experiences of women residing in other geographic areas within Pakistan. Likewise, due to the sample size and qualitative design of the current study, it is not possible to fully gauge the depth of these barriers across the selected communities, and other barriers may have been overlooked. Since our study focused on barriers to dietary intake, we did not specifically track micronutrient consumption during pregnancy. We suggest additional investigations across Pakistan designed to identify regional barriers to diverse and nutritionally dense diets among pregnant women and potential interventions.

## Conclusions

Adequate dietary intake during pregnancy is essential for both maternal and fetal health. Yet, restricted dietary intake during pregnancy is a common experience among women in Punjab, Pakistan. To explore the complexities of this occurrence, the present study used in-depth interviews and focus group discussions to uncover eight barriers that inhibited pregnant women from consuming nutritionally sound and diverse dietary intakes during pregnancy These were classified as physiological, socio-cultural and structural barriers. Our study reveals that in conjunction with making systemic changes at a structural level, much can be gained through implementing minor changes at the community, interpersonal and individual levels. For instance, the findings of our study could be used to raise greater awareness of the negative impact household food politics has on the health of both mother and child. As the nexus of dietary control within the household, specific campaigns can target mothers-in-law to alleviate dietary restrictions placed on pregnant mothers. Additionally, antenatal care that includes nutritional counseling may address the propagation of certain culturally validated, but potentially misguided, beliefs about diet during pregnancy. The negative impacts of cultural food classifications that prevent the consumption of protein-rich foods and certain fruits could be countered by narratives that link their consumption to essential micronutrients. Care that also addresses strategies to mitigate morning sickness, high blood pressure and unhealthy food cravings may also have a positive impact on maternal diet. Potentially, this process could be mediated through the active involvement of Lady Health Workers as part of outreach services that strengthen the nutritional status of pregnant women in rural areas. Furthermore, improving the accessibility of nutritious food options through low-cost initiatives, such as kitchen gardening, may provide pregnant mothers more opportunities to make healthy choices and consume healthful diets.

## Supplementary material

For supplementary material accompanying this paper visit https://doi.org/10.1017/S1368980021001737. click here to view supplementary material
