# Supplementary material for: What stops us from eating: a qualitative investigation of dietary barriers during pregnancy in Punjab, Pakistan
Source: Public Health Nutr. 2021 Apr 19;25(3):760–9. doi: 10.1017/S1368980021001737 (PMC8523588; doi:10.1017/S1368980021001737)
Supplement: Supplementary file 1 [file S1368980021001737sup001.pdf]

Supplementary material

What stops us from eating during pregnancy: A qualitative study in Punjab, Pakistan

Semi-structured guide for mothers

Hello, my name is Muhammad Asim. I am student at University of Sargodha, Pakistan. I am conducting a study to understand common eating practices among pregnant women and factors constraint consuming healthful food during pregnancy. We invited you to participate in this group discussion/individual interview because you have a child younger than 2 years of age. We want to learn from your knowledge and opinions toward dietary intake and food consumption pattern during pregnancy. The information that you will give us will help us to design a communication strategy to promote healthful eating habits for pregnant women in Punjab, Pakistan. Please keep in mind that there are no wrong answers. All answers are correct and feel free to ask any question during the interview.

I would also like to ask your permission to record the content of our discussions. I would like to record them because all of you will give us a lot of information and it can be difficult to capture all what you say in notes. This group discussion/individual interview will take approximately 60/30 minutes, respectively. The information that you will provide is confidential. Your name will not be revealed to anybody. The information that you will give us will be used to write a technical report. If you agree to participate, we can start now.

1. When you get pregnant what changes you observed in your diet?
    a. Did you start eating more or reduced the consumption of food?
    b. If reduce explore the causes.
    c. Probe about dietary behavior across the trimesters.
2. Probe about the individual preferences/carving/barriers of food intake during pregnancy.
    a. Probe about the reasons of craving about certain food items
3. Probe about diverse food choices and consumption patterns during pregnancy.
    a. Probe about the ideal diet during pregnancy and constraints to consume ideal diet.
4. How much changes occur in consumption of foods during pregnancy?
    a. Probe about the patterns of changes of food consumption

5. Probe about cultural beliefs/taboos about the different foods during pregnancy
6. Probe about the foods that are considered healthy during pregnancy.
    a. Why these foods are healthy?
    b. Who recommends these foods?
7. Probe about which foods are considered harmful during pregnancy?
    a. Why these foods are harmful?
    b. Who told these foods should not consume during pregnancy?
    c. Explore the food taboos during pregnancy.
    d. Probe about hot, cold, and hard foods that are voided during pregnancy.
8. Probe about the information and counseling received during pregnancy about dietary practices and food consumption.
9. Probe about the issues and opportunities to improve maternal diet and weigh gain during pregnancy.
10. Did health care providers ever counsel you about weight gain during pregnancy?
11. Explore the household and community level determinants of food intake patterns during pregnancy.
12. Who care your diet when you got pregnant at household/community level?

What stops us from eating during pregnancy: A qualitative study in Punjab, Pakistan

Semi-structured guide for healthcare providers

Hello, my name is Muhammad Asim. I am student at University of Sargodha, Pakistan. I am conducting a study to understand common eating practices among pregnant women and factors constraint consuming healthful food during pregnancy. We invited you to participate in this individual interview because you have been working as health care provider and have continuous interaction with pregnant women for counseling during antenatal visits. As a health care provider your perspective about the eating pattern of pregnant women would be helpful to understand dietary behavior of pregnant women from health care provider's perspective. Furthermore, your perspective will also helpful to understand and contextualize dietary behavior of pregnant women. The information that you will give us will help us to design a communication strategy to promote healthy eating habits for pregnant women in Punjab, Pakistan.

I would also like to ask your permission to record the content of our discussions. I would like to record them because all of you will give us a lot of information and it can be difficult to capture all what you say in notes. This interview will take approximately 30 minutes. The information that you will provide is confidential and we will use it for only research purpose. Your name will not be revealed and reported in any report. Your information only will be used to write a scientific report and your insight will be helpful to improve healthful eating behavior during pregnancy. If you agree to participate, we can start now.

1. Do eating habits of pregnant women change when they become pregnant?
    - Probe on how
    - Probe on whether pregnant women should eat the same amount of food as before, more, or less food during pregnancy.
2. What types of foods women wanted to eat during pregnancy?
3. Are there specific foods recommended for specific periods of pregnancy? (e.g., at the beginning of pregnancy, in the middle of pregnancy, around the end of pregnancy)
4. What are the reasons for recommending of these foods?
    - Probe on who recommends these foods.

5. Are there foods that are NOT recommended for pregnant women along specific periods of pregnancy? (Probe for hot, cold and hard concepts of foods)
6. In the household, who decides what pregnant women should eat?
7. Which are major factors that inhabit women consuming healthful diet during pregnancy?
    - Probe about the below barriers
    - Economic barriers
    - Physiological barriers
    - Individual barriers
    - Food meanings in diverse cultural settings
8. Are women receiving counseling about healthful diet during pregnancy?
9. How women will be able to consume healthful diet during pregnancy?
    - Initiate discussion for suggestions on how pregnant women diet can be strengthened at household, community and societal level
